# Inhibition of Castration-Sensitive LNCaP, Castration-Resistant TRAMP-C2, and Bone-Metastatic C4-2B Prostate Cancer Cell Growth by an Alpha-Tomatine Tomato Extract: In Vitro and In Vivo Study

**DOI:** 10.7759/cureus.102462

**Published:** 2026-01-28

**Authors:** Shunya Takeda, Miyu Uehara, Takashi Yurube, Shuji Ueda, Katsumi Shigemura

**Affiliations:** 1 Medical Engineering, Kobe University, Kobe, JPN; 2 Graduate School of Agricultural Science, Kobe University, Kobe, JPN; 3 Orthopaedic Surgery, Kobe University, Kobe, JPN; 4 Urology, Kurashiki Medical Center, Kurashiki, JPN

**Keywords:** alpha-tomatine (α-tomatine), c4-2b, castration-sensitive and castration-resistant prostate cancers, lncap, tomato extract, tramp-c2

## Abstract

Currently, hormonal therapy is the main treatment option for advanced prostate cancer; however, a certain number of cases progress to metastatic, castration-resistant prostate cancer. Therefore, we designed in vitro and in vivo studies of a new molecular targeted therapy using alpha-tomatine (α-tomatine), a glycoalkaloid extracted from tomatoes, for the growth inhibition of both castration-sensitive human LNCaP and castration-resistant mouse TRAMP-C2 and metastatic human C4-2B prostate cancer cell lines. In vitro, α-tomatine supplementation showed a dose-dependent decrease in the proliferation potential of all prostate cancer cells at concentrations ranging from 1.0 to 5.0 μg/mL, as well as a decrease in migration and invasion abilities at concentrations ranging from 1.0 to 2.5 μg/mL, which was sustained throughout the 72 hours post-treatment (p < 0.050). Furthermore, flow cytometry demonstrated that α-tomatine at 2.5 μg/mL enhanced the incidence of apoptosis in TRAMP-C2 cells at 48 hours post-treatment (p < 0.010). In vivo, TRAMP-C2 cells were subcutaneously implanted in C57BL/6 mice. Then, at a 10-mm diameter, single-time intratumoral injection of 1.0 µg/body α-tomatine was performed. Longitudinal follow-up identified a time-dependent tumor growth inhibition at 3-4 (p* *< 0.050), 5-7 (p < 0.010), and 8-10 (p < 0.001) days after the administration of α-tomatine. In summary, α-tomatine has the potential to block the proliferation, migration, and invasion of both castration-sensitive and castration-resistant prostate cancer (CRPC) types, even with metastatic cell lines. Further investigation is warranted to clarify the pharmacological action and molecular mechanism of α-tomatine’s effects on prostate cancer cells.

## Introduction

Prostate cancer is the most common cancer in Japanese men [1], and the second most frequent malignancy after lung cancer in men worldwide, developing 1,276,106 new cases and resulting in 358,989 deaths (3.8% of all deaths caused by cancer in men) in 2018 [2]. Prostate cancer is often asymptomatic in early stages, followed by complaints of non-specific difficulty with urination, increased frequency, and nocturia. In more advanced stages, urinary retention and back pain can occur, as the spine is the most common site of bone metastasis [3].

The standard treatment for prostate cancer localized to the gland is the combination of surgery, radiation therapy, and hormone therapy. In metastatic or recurrent disease, hormone therapy is the first-line treatment as well [4]. However, even with hormone therapy, approximately 20% of cases progress to castration-resistant prostate cancer (CRPC), which often becomes non-responsive within 18-24 months after the start of treatment [4]. In more recent years, the development of new molecular targeted therapies has improved the survival rate of CRPC patients; nevertheless, drug resistance is still a problem to be solved [4].

Alpha-tomatine (α-tomatine), a glycoalkaloid found in tomatoes, possibly serves as a defense against bacteria, fungi, viruses, and insects. Of nine tomato extracts, α-tomatine showed high inhibitory and anti-proliferative effects on all tested cell lines, including breast (MCF-7), colon (HT-29), gastric (AGS), and hepatoma (liver) (HepG2), as well as normal human liver cells (Chang) [5]. While the effectiveness of α-tomatine against hepatic HepG2 cells was greater than against colon HT-29 cells, the potencies of α-tomatine and alpha-chaconine at the concentration of 1 μg/mL against the liver carcinoma cell line were higher than those observed with anticancer drugs doxorubicin and camptothecin [6]. Regarding its anti-carcinogenic property, α-tomatine could inhibit 12-O-tetradecanoylphorbol 13-acetate (TPA)-induced adhesion, migration, and invasion, as well as the activation of extracellular signal-regulated kinase 1 and 2 (ERK1/2) and protein kinase C-alpha (PKC-α) involved in downregulating TPA-induced enzymatic activities, such as activation of nuclear factor kappa B (NF-κB) and messenger RNA [7].

Notably, human prostate cancer PC-3 cells were approximately 10 times more susceptible to inhibition by α-tomatine than human breast MDA-MB-231 and gastric KATO-III cancer cells and normal liver (Chang) and lung (Hel299) cell lines [8]. Moreover, the activity of α-tomatine against prostate cancer PC-3 cells was 200 times greater than that of the aglycone tomatidine [8].

The exposure to glycoalkaloids produced by eggplants (alpha-solamargine and alpha-solasonine), potatoes (alpha-chaconine and alpha-solanine), and tomatoes (α-tomatine) or their hydrolysis products (monosaccharide, disaccharide, and trisaccharide derivatives and the aglycones solasodine, solanidine, and tomatidine) inhibits the growth of cancer cells [9]. The tomatidine, the aglycone of α-tomatine abundant in green tomatoes, significantly inhibited palmitate-provoked lipid accumulation and stimulated the phosphorylation of adenosine monophosphate-activated protein kinase (AMPK) and acetyl-CoA carboxylase 1 (ACC1) in human HepG2 hepatocytes, clarifying that tomatidine functions as an agonist for vitamin D receptor to elicit AMPK-dependent suppression of lipid accumulation [10]. The inhibition of human breast adenocarcinoma MCF-7 cell proliferation and viability by α-tomatine at concentrations of 6 and 9 µM also resulted from the loss of ATP but without any signs of apoptosis induction [11].

The purpose of this study was to investigate the effects of α-tomatine on prostate cancer cell growth in vitro and in vivo and to evaluate its potential as a novel molecular targeted therapy for advanced CRPC, including cell lines with bone metastasis.

## Materials and methods

Cells

Human prostate cancer cell lines LNCaP [12] and C4-2B [13] and a mouse prostate cancer cell line TRAMP-C2 [14] were used, as shown in our previous papers [15,16]. The LNCaP is castration-sensitive, whereas C4-2B is an established epithelial cell line to study the development of metastatic CRPC. TRAMP-C2 is an epithelial cell line derived from the transgenic castration-resistant adenocarcinoma of an adult male C57BL/6 mouse prostate model. While LNCaP and C4-2B were cultured in the medium (RPMI-1640; Sigma-Aldrich, St. Louis, MO, USA), Dulbecco’s Modified Eagle’s Medium (D5796, Sigma-Aldrich) was used for TRAMP-C2 under 5% CO_2_ conditions at 37 °C [17,18]. Both media contained 10% fetal bovine serum (F2442, Sigma-Aldrich) with 1% penicillin and streptomycin (26253-84, Nacalai Tesque, Kyoto, Japan).

Treatments

Alpha-tomatine (CAS No. 17406-45-0; Tokyo Kasei Kogyo, Tokyo, Japan), a compound derived from tomatoes, was used in this study. Alpha-tomatine was dissolved in dimethyl sulfoxide (DMSO; CAS No. 13406-55; Nacalai Tesque, Kyoto, Japan). Administration of the DMSO vehicle alone at the same dose served as the control intervention.

Cell proliferation assay

The LNCaP, C4-2B, and TRAMP-C2 cells were seeded in a 96-well plate at 1.0 × 10^4^/well (n = 3). Twenty-four hours later, the initial medium was replaced with a medium containing 1.0-5.0 μg/mL α-tomatine. Cells experiencing the change to the medium containing only DMSO at the same concentration were used as the control group. Cell proliferation based on the dehydrogenase activity of Cell Counting Kit-8 (CK04, Dojindo Laboratories, Kumamoto, Japan) was calculated by the plate reader with the absorbance at 450 nm, over time (0, 24, 48, and 72 hours) [6].

Wound-healing assay

The LNCaP, C4-2B, and TRAMP-C2 cells were seeded in a 12-well plate at 1.0 × 10^5^/well (n = 3). Following 80%-90% confluency, a single cell layer of each well was wounded using a 200-µL pipette tip, and the medium was replaced with medium containing 1.0-2.5-μg/mL α-tomatine [14]. Similarly, the control group received medium containing DMSO only. Cells were monitored using the BZ-X700 microscope (Keyence, Osaka, Japan) over time (24, 48, and 72 h) [7].

Apoptosis assay

The TRAMP-C2 cells were seeded in a 6-well plate at 1.0 × 10^6^/well (n = 3). Then, a 24-h culture was followed by the replacement with a medium containing 1.0-2.5 μg/mL α-tomatine or DMSO control. After an additional 48-h culture, in flow cytometry with the Annexin V-FITC Apoptosis Detection Kit (Nacalai Tesque), early (green signal by Annexin V-FITC) and late (red signal by propidium iodide) apoptotic cells were detected using the CytoFLEX S flow cytometer (Beckman Coulter, Brea, CA, USA).

Animals

Eight-week-old male/female C57BL/6 mice (CLEA Japan, Tokyo, Japan) were used (total: n = 8). The TRAMP-C2 cells were dissolved in the Matrigel matrix (356234, Corning Inc., Corning, NY, USA) and subcutaneously implanted. When the tumor diameter reached 10 mm, mice were divided into the treatment and control groups. Randomization was performed to ensure that the average body weight and tumor size were similar between the treatment and control groups. While the single-time intratumoral administration of 1.0 µg/body α-tomatine was performed in the treatment group, intratumoral DMSO injection was conducted in the control group (each n = 4). Then, the tumor diameter was measured daily up to 10 days after administration, and the tumor volume was measured as previously described [19]. This experimental procedure was performed according to the regulations of the Institutional Animal Care and Use Committee (approval number: P230906; approval date: September 21, 2023) at Kobe University Graduate School of Medicine, Kobe, Japan, and conducted in accordance with the laws and regulations of Japan.

Statistical analysis

Descriptive statistics for continuous variables are presented as means ± standard deviation (SD). In vitro, data were obtained technically in duplicate (two technical replicates). All experiments were independently performed with at least three biological replicates (n = 3). In vivo, experiments were independently conducted with eight biological replicates because of the individual animal differences (n = 8). Following the normality assumption, mixed-design ANOVA with the Tukey-Kramer post-hoc test was used. All statistical tests with statistical significance set at p-values of <0.050, <0.010, and <0.001 were performed using IBM SPSS Statistics for Windows, Version 28.0 (Released 2021; IBM Corp., Armonk, NY, USA).

## Results

In vitro cell proliferation affected by α-tomatine

**Figure 1:**
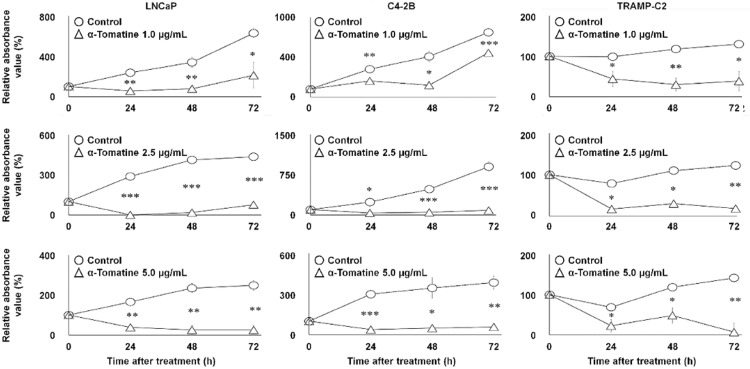
In vitro time-course changes in the absorbance of LNCaP, C4-2B, and TRAMP-C2 cell viability for 0-72 h after 1.0-5.0 μg/mL α-tomatine treatment. Data were obtained in duplicate (two technical replicates) and are shown as mean ± SD (three biological replicates). Comparisons were made between α-tomatine-treated (triangle) and DMSO control (circle) groups (each n = 3). Two-way repeated measures ANOVA with the Tukey-Kramer post-hoc test was used. *p < 0.050. **p < 0.010. ***p < 0.001.

Figure 1 shows the time-course changes in absorbance for the treatment and control groups following α-tomatine administration. In all tested cell lines, α-tomatine significantly inhibited proliferation at 1.0-5.0  µg/mL after 24 hours (p < 0.05).

In vitro wound healing affected by α-tomatine

**Figure 2:**
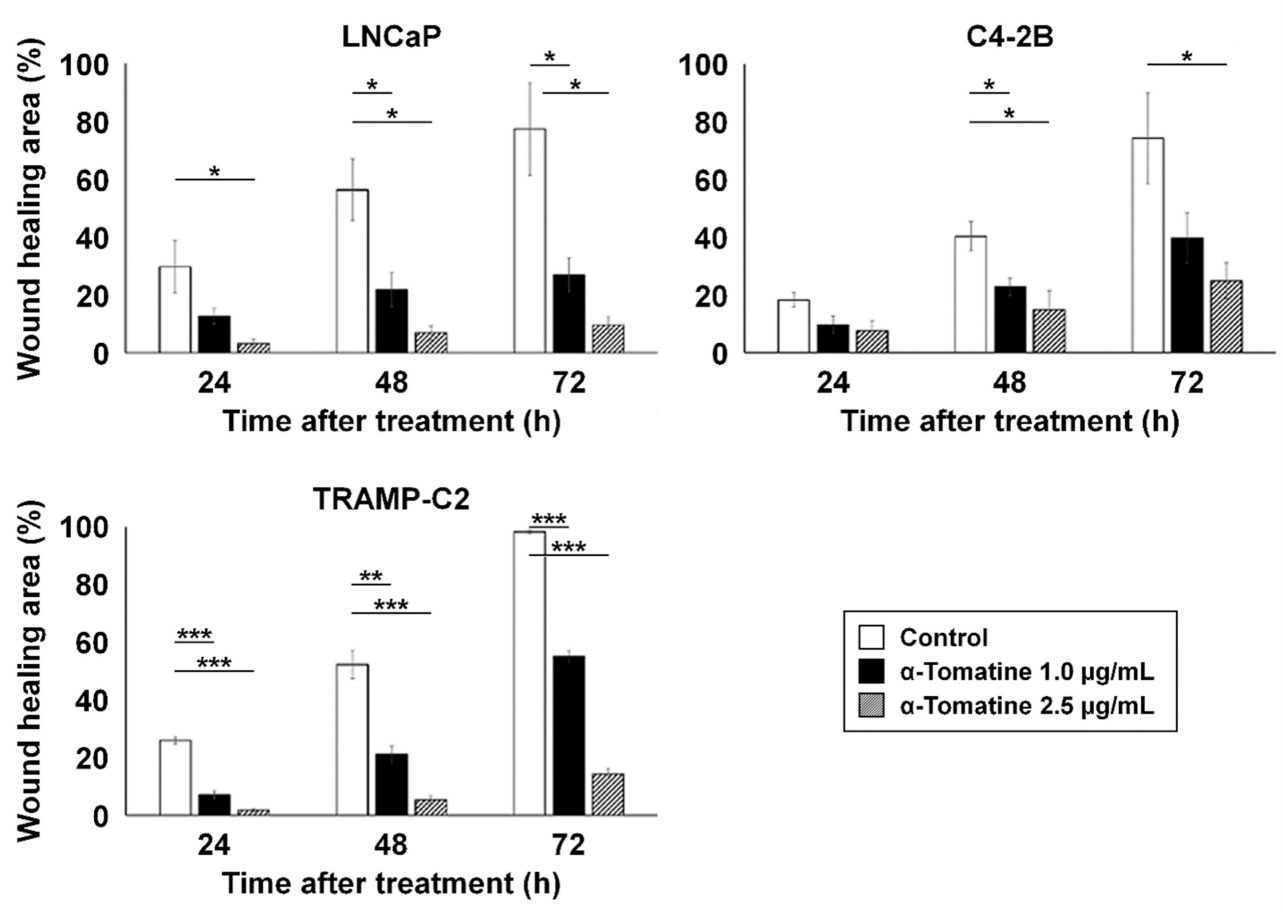
In vitro time-course changes in wound-healing area (%) of LNCaP, C4-2B, and TRAMP-C2 cells at 24-72 hours following treatment with 1.0 and 2.5 µg/mL α-tomatine. Data were obtained in duplicate (two technical replicates) and are shown as mean ± SD (three biological replicates). Comparisons were made among the 1.0 μg/mL α-tomatine-treated (black), 2.5 μg/mL α-tomatine-treated (gray), and DMSO control (white) groups (each n = 3). Two-way repeated measures ANOVA with the Tukey-Kramer post-hoc test was used. *p < 0.050. **p < 0.010. ***p < 0.001.

Figure 2 illustrates the temporal changes in wound-healing area (%) after wounding. In LNCaP cells, α-tomatine at 1.0 and 2.5 µg/mL significantly inhibited wound healing after 48 and 72 hours (p < 0.05). Similar inhibition was observed in C4-2B cells at 48 hours (p < 0.05). TRAMP-C2 cells also showed significant suppression of wound-healing capacity at 24, 48, and 72 hours (p < 0.01).

In vitro apoptosis affected by α-tomatine

**Figure 3:**
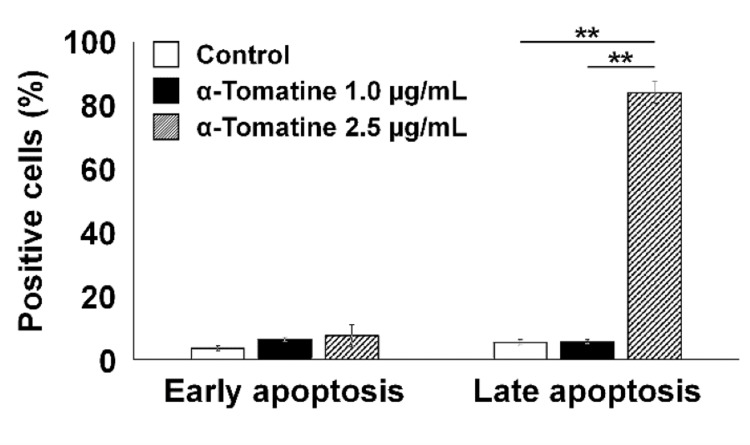
In vitro changes in the percentage of early and late apoptotic TRAMP-C2 cells 48 hours after treatment with 1.0-2.5 µg/mL α-tomatine. Data were obtained in duplicate (two technical replicates) and are presented as mean ± SD (three biological replicates). Comparisons were made between 1.0 µg/mL α-tomatine (black), 2.5 µg/mL α-tomatine (gray), and DMSO control (white) groups (each n = 3). Early and late apoptotic cells were measured separately. One-way repeated measures ANOVA with Tukey–Kramer post-hoc test was applied. **p < 0.010.

As shown in Figure 3, in TRAMP-C2 cells, treatment with 1.0 µg/mL α-tomatine did not significantly alter the percentage of apoptotic cells compared to the control. Similarly, in the 2.5 µg/mL α-tomatine group, the proportion of early apoptotic cells was comparable to the control, showing no significant difference. However, the 2.5 µg/mL group exhibited a significantly higher percentage of late apoptotic cells compared with both the 1.0 µg/mL α-tomatine and control groups (p < 0.010), despite early apoptosis remaining unchanged.

In vivo tumor growth affected by α-tomatine

**Figure 4:**
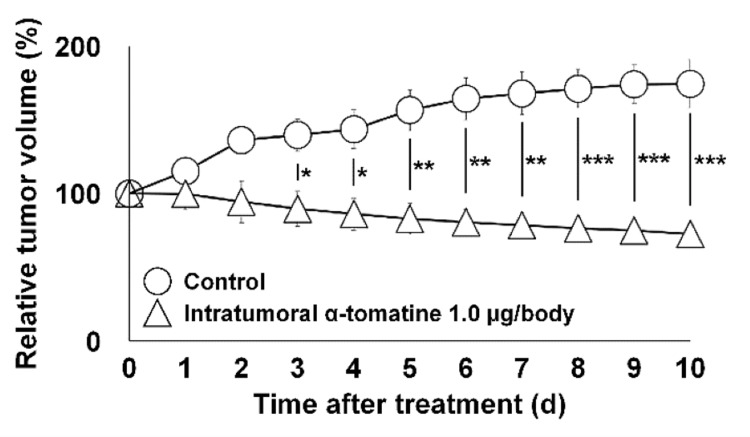
In vivo time-course changes in TRAMP-C2 tumor volume over 0–10 days in mice after a single intratumoral injection of 1.0 µg/body α-tomatine. Data are presented as mean ± SD (n = 4 biological replicates per group). Comparisons were made between the α-tomatine-treated group (triangle) and the DMSO control group (circle). *p < 0.050. **p < 0.010. ***p < 0.001.

Figure 4 shows the longitudinal changes in tumor volume relative to baseline (pre-treatment set as 1) in the α-tomatine-treated and DMSO control groups. While no significant differences were observed in general condition, body weight, or mortality between groups, α-tomatine treatment produced significant tumor growth inhibition starting from day 3 of administration (p < 0.050).

## Discussion

In this study, the α-tomatine-treated group showed significant inhibition of cell proliferation in castration-sensitive LNCaP cells as well as castration-resistant C4-2B and TRAMP-C2 cells. The α-tomatine-treated group also exhibited a significantly higher percentage of apoptotic TRAMP-C2 cells. In animal experiments, the rate of tumor volume increase was significantly lower in the drug-treated group, suggesting that α-tomatine effectively inhibits the growth of prostate cancer cell lines. Additionally, α-tomatine prevented wound healing in LNCaP, C4-2B, and TRAMP-C2 cells, suggesting suppression of migration and invasion in these prostate cancer cell lines. Based on these findings, α-tomatine may have the potential to inhibit proliferation, migration, and invasion in both castration-sensitive and castration-resistant prostate cancer cells, including metastatic forms.

Accumulating evidence indicates that α-tomatine exerts its effects through multiple mechanisms, including inactivation of the intracellular phosphatidylinositol 3-kinase (PI3K)/Akt and ERK signaling pathways [20,21], inhibition of transcription factors NF-κB, c-Fos, and c-Jun [20,22-24], and reduction of matrix metalloproteinases (MMP-2 and MMP-9) and urokinase-type plasminogen activator (u-PA) activities [20,25]. These mechanisms may contribute to its anti-metastatic effects against prostate cancer [20].

Specifically, combined treatment with a subtoxic dose of α-tomatine and paclitaxel significantly decreased cell viability and increased apoptosis in the PC-3 prostate cancer cell line. This effect was associated with inhibition of PI3K/Akt signaling, decreased levels of anti-apoptotic proteins Bcl-2 and Bcl-xL, and increased levels of pro-apoptotic protein BAD [21].

Lycopene, a carotenoid with high antioxidant capacity extracted from tomatoes, is also effective in prostate cancer prevention and treatment through PI3K/Akt signaling, as it accumulates at higher concentrations in prostate tissue compared to other organs [26].

Furthermore, α-tomatine exhibits strong anti-cancer effects, particularly against CRPC, as well as robust anti-fungal activity, through modulation of the NF-κB/ERK signaling pathway [22]. Among α-tomatine, β1-tomatine, γ-tomatine, δ-tomatine, and tomatidine, α-tomatine was the most effective in reducing tumor necrosis factor-alpha (TNF-α) and inhibiting cancer cell growth [8].

Alpha-tomatine has been shown to inhibit phosphorylation of Akt and ERK1/2 in human lung adenocarcinoma A549 cells and downregulate MMP-2, MMP-9, and u-PA expression [20]. It also reduces nuclear levels of NF-κB, c-Fos, c-Jun, and AP-1 [20]. These findings suggest that α-tomatine may act as an anti-metastatic agent against human lung adenocarcinoma. However, there are no published studies regarding α-tomatine’s inhibition of metastasis in prostate cancer. To our knowledge, this is the first report suggesting potential anti-metastatic effects of α-tomatine, demonstrated by marked suppression of metastatic castration-resistant C4-2B cells.

Exploiting the anti-inflammatory and anticancer properties of curcumin, a polyphenol extracted from turmeric rhizomes [25], combination therapy of α-tomatine with curcumin strongly inhibited growth and induced apoptosis in human prostate cancer PC-3 cells [27].

We would like to acknowledge the limitations of this study. First, additional mechanistic assays, particularly in vitro, are needed to detail the apoptotic cascade. Second, animal studies require immunohistochemical analyses with additional human cell lines (such as PC-3) to elucidate the mechanisms underlying α-tomatine’s anti-tumor effects. Third, comparative studies with other forms of tomatine, such as β-tomatine, would be informative. Fourth, we did not investigate key signaling pathways, including pro-survival NF-κB, anti-apoptotic proteins like Bcl-2, the PI3K/Akt pathway, and ERK1/2, nor mechanisms of caspase-independent apoptosis. Additionally, studies using normal prostate cells (RWPE-1) were not conducted. Finally, further assays, including Cell Counting Kit-8 (CCK-8) and Annexin V-FITC, are needed for more precise analysis. Future studies will clarify the molecular mechanisms by which α-tomatine inhibits cancer cell proliferation, migration, and invasion and induces apoptosis.

## Conclusions

In our in vitro study, α-tomatine significantly inhibited the growth of prostate cancer cells and suppressed their migration and invasion. Notably, in our in vivo study, the main feature of this work, local administration of α-tomatine significantly reduced tumor growth. Although further mechanistic studies are needed, these findings suggest a potential strategy for prostate cancer prevention. Additional research is warranted to elucidate the pharmacological actions and molecular mechanisms underlying α-tomatine’s effects on prostate cancer cells.
